# Scalable production of bio-calcium oxide via thermal decomposition of solid - hatchery waste in a laboratory-scale rotary kiln

**DOI:** 10.1038/s41598-024-84889-w

**Published:** 2025-01-05

**Authors:** Suwanan Chuakham, Ajchara I. Putkham, Yuwadee Chaiyachet, Arnusorn Saengprajak, Kriangsak Banlue, Nipon Tanpaiboonkul, Apipong Putkham

**Affiliations:** 1 Department of Environmental Technology, Faculty of Environment and Resource Studies, Mahasarakham University, Mahasarakham, 44150 Thailand; 2 Department of Chemistry, Faculty of Science, Naresuan University, Phitsanulok, 65000 Thailand; 3 Department of Physics, Faculty of Science, Mahasarakham University, Mahasarakham, 44150 Thailand; 4 Department of Food Technology and Nutrition, Faculty of Technology, Mahasarakham University, Mahasarakham, 44150 Thailand

**Keywords:** Circular economy, Waste utilization, Renewable materials, Eggshell, Catalyst, Green chemistry, Sustainability, Materials chemistry, Chemical synthesis, Porous materials, Ceramics

## Abstract

Chicken eggshell waste is an alternative renewable source for quicklime production. Eggshell waste has received significant attention from researchers due to it being a potential source of bio-CaO, which not only drives the circular economy concept but also supports sustainable development. However, experiments on the production of bio-CaO are normally conducted in a small lab-scale furnace. Furthermore, the eggshell raw material is collected from canteens or households, which is not suitable for economical or industrial production. Therefore, this study investigated the factors affecting the bio-CaO production from hatchery waste via both batch and continuous calcination process in a laboratory-scale rotary kiln for the first time. The eggshells were first separated from the solid hatchery waste. Then, the effect of preparation methods of raw eggshells on the properties of bio-CaO was investigated, including eggshells with and without membrane separation, various particle sizes, and with an increase of the percent raw material filling in the kiln from 5 to 20%. Calcination of the samples was performed in a rotary kiln at 800 °C with a 0.5 RPM rotating speed and a 5° inclination of the kiln. The effects of the calcination process in either an air or N_2_ atmosphere on the calcined product were also observed. Instrumental analysis shows that the production yield and purity of bio-CaO were in the range of 49–56 wt% and 97–98%, respectively. The results also indicated that the production yield of bio-CaO decreased to 17.7% with a decrease in the raw material particle size from 3.3 mm to 250 μm. Moreover, the production of bio-CaO with eggshells containing eggshell membrane decreases the purity of calcium oxide by about 0.7–1.0%. In addition, further increasing the filling volume of the kiln from 5 to 20% had only a slight effect on the purity and yield of the product. These results imply that it is not necessary to remove the eggshell membrane from the raw eggshells in order to produce industrial-grade CaO from the raw eggshell. These new findings can likely be used to develop an alternative process design to reduce the manufacturing cost of bio-CaO produced from hatchery waste. Furthermore, this present study reveals that the specifications of the obtained bio-CaO comply with both Thai industrial standards and international food additive standards.

**Supplementary Information:**

The online version contains supplementary material available at 10.1038/s41598-024-84889-w.

## Introduction

Calcium oxide or quicklime is one of the most versatile chemicals used in both research and industrial applications such as industrial catalyst/filler, food/cosmetic additive, medical treatment, carbon dioxide capture, and environmental remediation^1–4^. The process of quicklime production is associated not only with exploitation of geo-resources but also involves both local and global scale environmental impact such as emissions of large quantities of both fine particle dust and CO_2_.More importantly, the world production of quicklime is responsible for around 8% of global anthropogenic CO_2_ emissions^5^. Shan et al. reported that 69% of CO_2_ emission from Chinese industrial sector is related to lime production^6^. Life cycle analysis indicated that limestone quarterly process in Thailand includes basting, transportation, and crushing/grinding and emits about 3.13 kg CO_2_ eq. per ton of limestone rock product^7^. As a result, research aimed at finding sustainable raw materials for partial or total substitution of natural lime application has been reported using various starting materials for example seashells, avian eggshells, and waste containing calcium oxide^8,9^. Among these alternative raw materials, chicken eggshells have attracted considerable attention. This is likely because (1) high purity calcium oxide can be obtained at a calcination temperature of 800 °C, which is lower than for other shells^10–12^, and (2) chicken eggshell waste isa cheap alternative. Furthermore the significant daily consumption of the eggs makes eggshell waste available in domestic sector household, canteen, and restaurant^13^. However, one major obstacle to the progression of industrial production of calcium oxide from eggshells is the daily collection of the eggshell waste from each household and canteen to a recovery facility, which can potentially lead to high operating costs of the industrial production.

Alternatively, the in-situ production of bio-calcium oxide derived from chicken hatchery industry is possibly a more practical approach. In Thailand, a chicken hatchery farm normally generates solid hatchery waste in the range of 0.5-2.0 tons per day^11,14^. In addition, the potential amount of solid hatchery waste produced in Thailand is approximately about 876,000 ton per year. This solid hatchery waste is comprised from eggshells, eggshell membrane, dead chickens, and a viscous liquid from eggs and decaying tissue. The solid hatchery waste is usually disposed into landfills and causes both environmental impact and conflict with surrounding communities. Thus, valorization of hatchery eggshell waste is a priority for achieving circular economy while simultaneously reducing industrial production costs. Furthermore, recycling of hatchery eggshell waste as a calcium oxide can also reduce the risk of microbiological contamination in the environment^14^. Chicken eggshell is composed from calcium carbonate (94–97%) while the remainder is organic matter and trace elements. Beside this the eggshell membrane contains about 85–90% of protein and 10–15% of trace inorganic element. The densities of the eggshell and outer eggshell membrane have been reported in the range of 2.01–2.62 g/cm^3^ and of 1.36 g/cm^3^, respectively^15,16^. These variations of the amount of calcium carbonate and density of the eggshell may depend on the species and age of the chicken as well as on the supplied food^17^. Our preliminary study showed that direct substitution of hatchery eggshell waste for natural lime stone in cement production process leads to detrimental effect on the cement product^18^. This indicates that high purity bio-calcium oxide derived from eggshell is required for some production processes. Production of eggshell derived calcium oxide depends on two main processes which are (1) pretreatment process and (2) conversion process. The objective of the pretreatment process is to remove all impurity materials adhered to the eggshell waste such as dirt and eggshell membrane, and possibly to result in size reduction. Household eggshell waste is normally treated by washing with tap water followed by drying. Then, the membrane is removed by hand. However, several researchers proposed that there are three feasible industrial production techniques for membrane removal. The first possibility is heat treatment at the temperature in the range of 300–500 °C^12,19^. The second option is chemical treatment by reagents such as EDTA, chlorine, and hydrochloric or acetic acid^20–23^. The third separation technique is related to physical processes such as floatation or using a centrifugal separator. There are also different approaches for the conversion process. For example, special property eggshell calcium oxide such as nano-calcium oxide can be produced by chemical precipitation, ball milling, or sol-gel techniques^24^. Normally, thermal conversion is the conventional technique used for decomposing the eggshells to calcium oxide and carbon dioxide. Nevertheless, thermal conversion technique has some advantages such as no chemicals are being used in the process and less waste management is needed. As mentioned earlier, calcination of eggshells at 800 °C for 1 h is adequate for obtaining calcium oxide with purity in the range of 97–98%.However, extending calcination time beyond 1 h can probably result in the decrease of both surface area and pore size of calcium oxide^25^. It should also be noted that research works involving eggshell calcination are usually done using a small bench top muffle furnace or a large volume muffle furnace. However, these muffle furnaces have some disadvantages. For example, a muffle furnace is conventionally run in a batch operation and there is no mixing or agitation mechanism in the furnace. This can potentially lead to insufficient heat induction and convection for completing the thermal decomposition of a large amount of eggshell waste. Putkham et al.^26^ reported that calcination of a large amount of eggshell waste in an industrial car-bottom furnace is not efficient for completely converting the eggshells to calcium oxide. This is probably due to the batch operation in the car bottom furnace not being able to provide uniform heating to the whole amount of eggshell waste. Thus, a furnace with a mixing mechanism or equipment is required.

Rotary kilns have been commercialized for decades, especially in cement production, incineration of hazardous wastes, and for biomass pyrolysis. In contrast to other types of furnaces, the rotary kiln offers some unique advantages over the muffle furnace or car bottom furnace. For example, the slow rotational speed of the inclined kiln enables thorough mixing of raw materials. Also, the residence time of raw materials can be easily adjusted to provide the optimum conditions for the thermal reaction. Additionally, various shapes and sizes of the raw material can be fed into a rotary kiln either in batches or continuously^27,28^. However, a thorough search of the relevant literature yielded only one related article on the performance of rotary kiln reactors for shell calcination. Barros et al.^9^ proposed a comprehensive industrial process for calcination of mussel shell to calcium carbonate using a 17 m long rotary kiln with a 2.5 m inner diameter. The operating calcination conditions were 600 °C with 2 rpm and solid resident time of 20–30 min. This process yielded calcium carbonate output of about 70–80%wt of the mussel shell input. Unlike other studies, factors effecting the production of high purity bio-calcium derived from calcination of eggshell waste in a laboratory-scale rotary furnace is reported for the first time in this study. The influence of preparation methods of solid hatchery waste including particle size, membrane removal, different calcination atmosphere, and material feeding rate on the properties of the calcium oxide product are systematically described. Furthermore, for multipurpose applications of bio-calcium oxide as a filler, the properties of these obtained bio-calcium oxide were compared with both food and industrial standards.

## Exerimental section

### Materials

Hatchery solid waste was collected from a large broiler hatchery farm in Nakhon Ratchasima province, northeast of Thailand. This hatchery farm produces around 1.0-1.5 tons of hatchery solid waste daily. An industrial grade—quicklime (≥ 90%) was obtained from Lime Master Co., Ltd., Thailand. Acetic acid (37%) and Calcium oxide were (≥ 97%) obtained from RCI Labscan Ltd and Daejung Chemicals, respectively. The two commercially available CaO samples, industrial-grade quick lime and laboratory grade, were used as reference materials and for comparison with the eggshell waste derived samples. All commercially available chemicals utilized in this study were used as supplied without any further purification.

### Preparation of eggshell samples

**Fig. 1:**
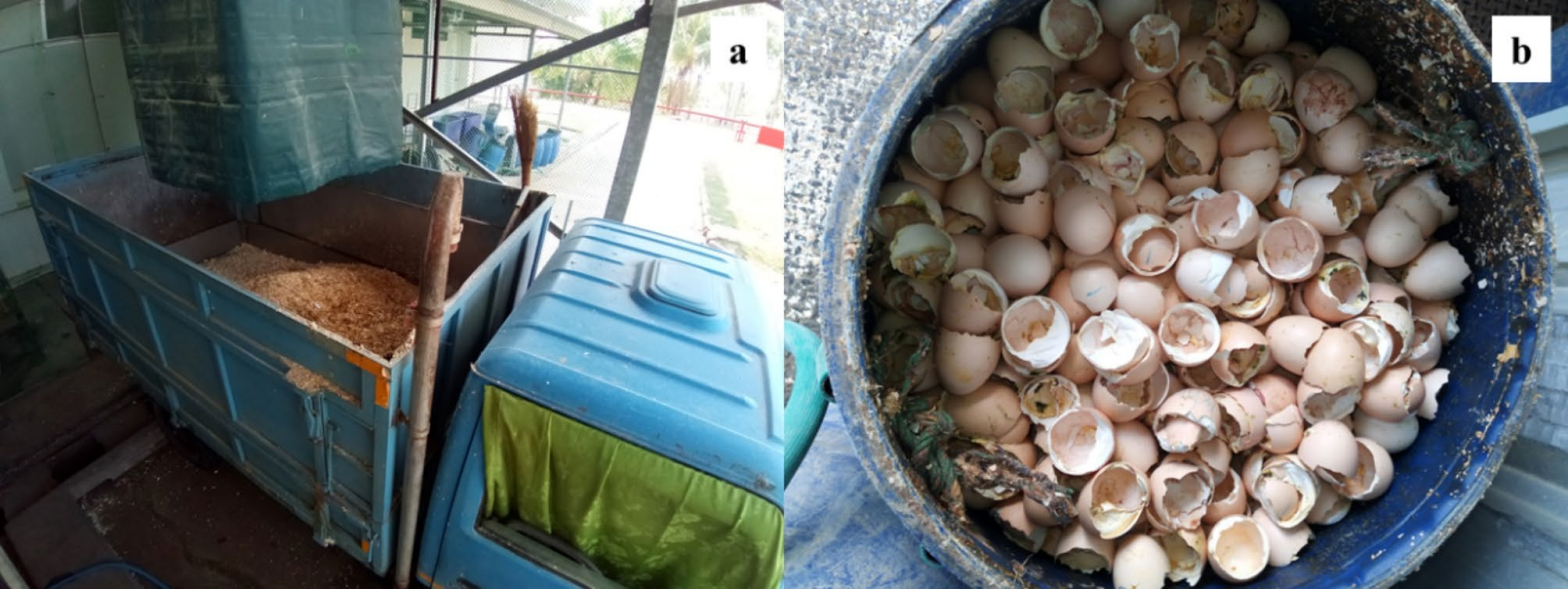
Separated eggshell waste generated daily at a hatchery farm.

**Fig. 2:**
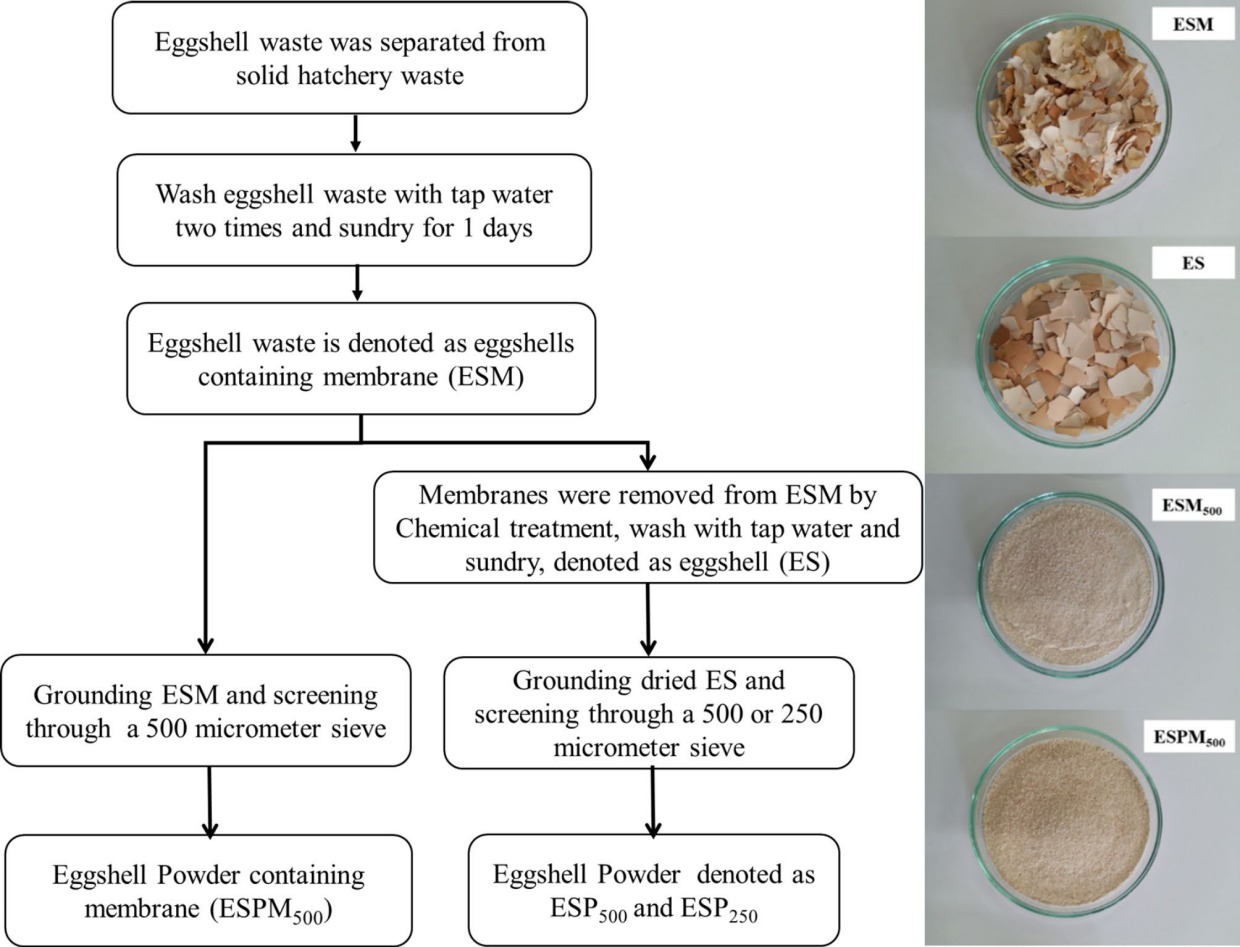
Preparation of eggshell samples and photos of the samples after preparation.

The following four preparation methods were employed to obtain four different eggshell samples for calcination: (1) Eggshell: ES, (2) Eggshells containing membrane: ESM, (3) Eggshell powder: ESP, and (4) Eggshell powder containing membrane: ESPM. The preparation methods are summarized as follows. Initially, solid hatchery waste produced from the farm was routinely passed through a screw conveyor and manual sorting to separate dead embryos from chicken eggshell waste as shown in Fig. 1. Then the separated eggshell waste was thoroughly washed twice with tap water to remove the viscous liquid adhered to the solid hatchery waste. Subsequently, the washed eggshell waste was sundried for 1 day. It should be noted that the eggshell waste must be cleaned to remove the viscous liquid and dried otherwise a highly odorous ammonium compound will be formed during the eggshell calcination process. The sieve analysis revealed that the sundried eggshell waste comprises of both eggshells (95.6 ± 2.2% wt) and eggshell membranes (4.4 ± 1.3%wt) with an effective particle size of 3.3 mm (D_60_) and coefficient of uniformity (UC) of 2.64, which means that the sundried eggshell waste has a narrow range of particle sizes. Additionally, this sundried eggshell waste was denoted as eggshells containing membrane (ESM). Combined chemical and mechanical treatments were used for removing the eggshell membrane from the ESM sample and brief description of this combined treatment is as follows. Firstly, 10 kg of the ESM material was impregnated in a 150 l stainless steel reactor containing 0.1 M acetic acid. The spiral propeller blades in the reactor were operated at 100 rpm for 30 min to homogenize the sample in the weakly acidic solution. Then, the acid solution was drained out and the ESM samples were placed in another reactor equipped with an 1 HP aerator. The floating eggshell membranes were drained out while the eggshell waste, which settled at the bottom of the floatation reactor, was collected, and washed with tap water and sundried again for 1 day. This sample was denoted as eggshell (ES). Additionally, both ESM and ES were ground with Panasonic MX-AC400 grinding machine followed by screening through either a 500-µm sieve (No. 35) or a 250-µm sieve (No. 60) to obtain the powder of eggshells containing membrane (ESPM) and eggshell (ESP), which are denoted with the suffix 500 or 250 to identify their size (for example, ESPM_500_ andESPM_250_). The whole eggshell waste preparation method and photos of the samples after preparation are shown in Fig. 2.

### Calcination of eggshell waste in a laboratory-scale rotary kiln

**Fig. 3:**
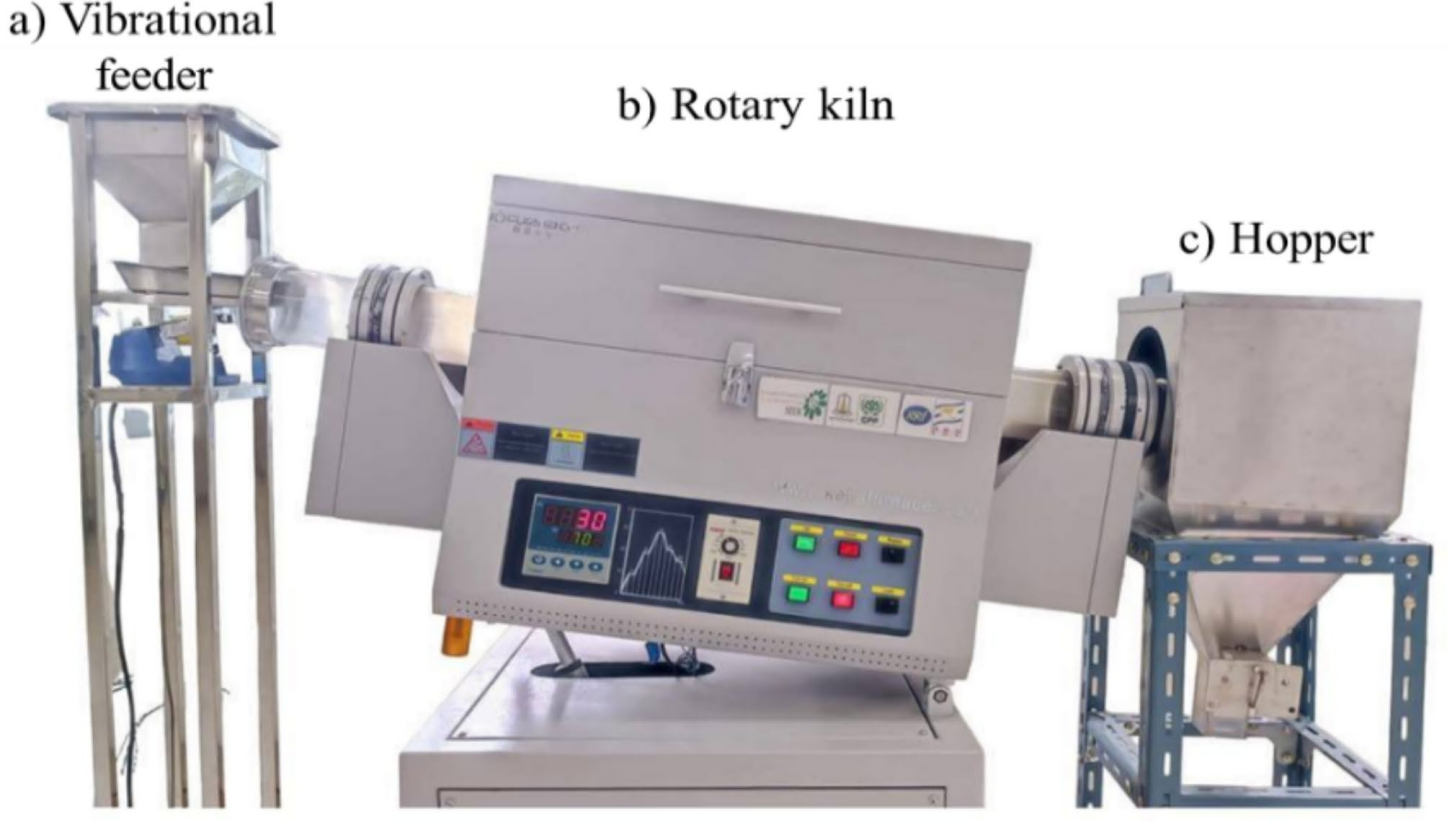
Photo of a laboratory-scale rotary kiln reactor consisted of (**a**) vibrational feeder, (**b**) rotary kiln, and (**c**) hopper.

Calcination of different samples of eggshell waste (later denoted with the prefix -C for example, CES) was carried out using a laboratory-scale rotary kiln as shown in Fig. 3. This indirectly heated rotary kiln chamber was made of a quartz tube with an inner diameter of 80 mm and a 100 mm outer diameter. The effective heated length of the reactor was approximately 440 mm, and the overall length was 1200 mm. The kiln had no lifters installed. A set of 6 kW PID- controlled heaters was used to heat the kiln. The temperature at the electrical heater is measured directly using a type K-thermocouple. Before conducting the experiments, the actual temperature distribution inside the kiln chamber is measured directly using thermocouples to ensure that temperature at the effective heated zone of the quartz tube was set to be about 810 ± 4.2 °C as shown in supplementary data. For entire experiments, the kiln was set to be 5° inclination with a constant rotating speed of 0.5 rpm. In this circumstance, the residence time of the samples in the heating zone of the rotary kiln was calculated from a conventional equation^27,29^ is ~69 min. The calcination was carried out under the atmosphere of either air or N_2_. In this study, both batch and continuous calcination experiments were conducted in order to determine the effect on scalable production of CaO.

For the first batch experiment, 0.26 kg of the eggshell samples (which is about 5% of the effective volume of the kiln) prepared by different methods were then fed into the entrance of the rotary kiln via a vibrational feeder to determine the effect of the preparation method on the properties of the obtained bio-CaO products. After the calcined product flow through the kiln to the hopper, the products were then mixed and kept in desiccator for characterization. For the second batch experiment, the same calcination experiment as described above was conducted. However, this time the samples were calcined under N_2_ atmosphere instead of the air atmosphere. Abbreviation of the calcined samples obtained under N_2_ atmosphere includes the suffix –N_2_.

For the continuous calcination experiment, the sample obtained from the optimum preparation method, which was chosen from the first batch experimental setup, was then fed into the kiln with different feeding rate to determine the effect of raw material filling rate into the rotary kiln on the properties of the bio-CaO. The feeding rate was increased from 0.26 kg/h (5% of the kiln effective volume) to 0.51 kg/h and 1.03 kg/h, which corresponds to 10% and 20% of the effective volume of the kiln, respectively. Several research reports suggest the optimum degree of particle filling in the kiln. Karunarathne et al.^29^ pointed out that the optimum degree of biomass filling in the furnace is 20–25%. In addition, Niessen^29^ reported that degree of waste filling in the rotary incinerator should not excess 20%. A high degree of material filling normally causes low heat transfer efficiency and leads to incomplete calcination of the product.

**Fig. 4:**
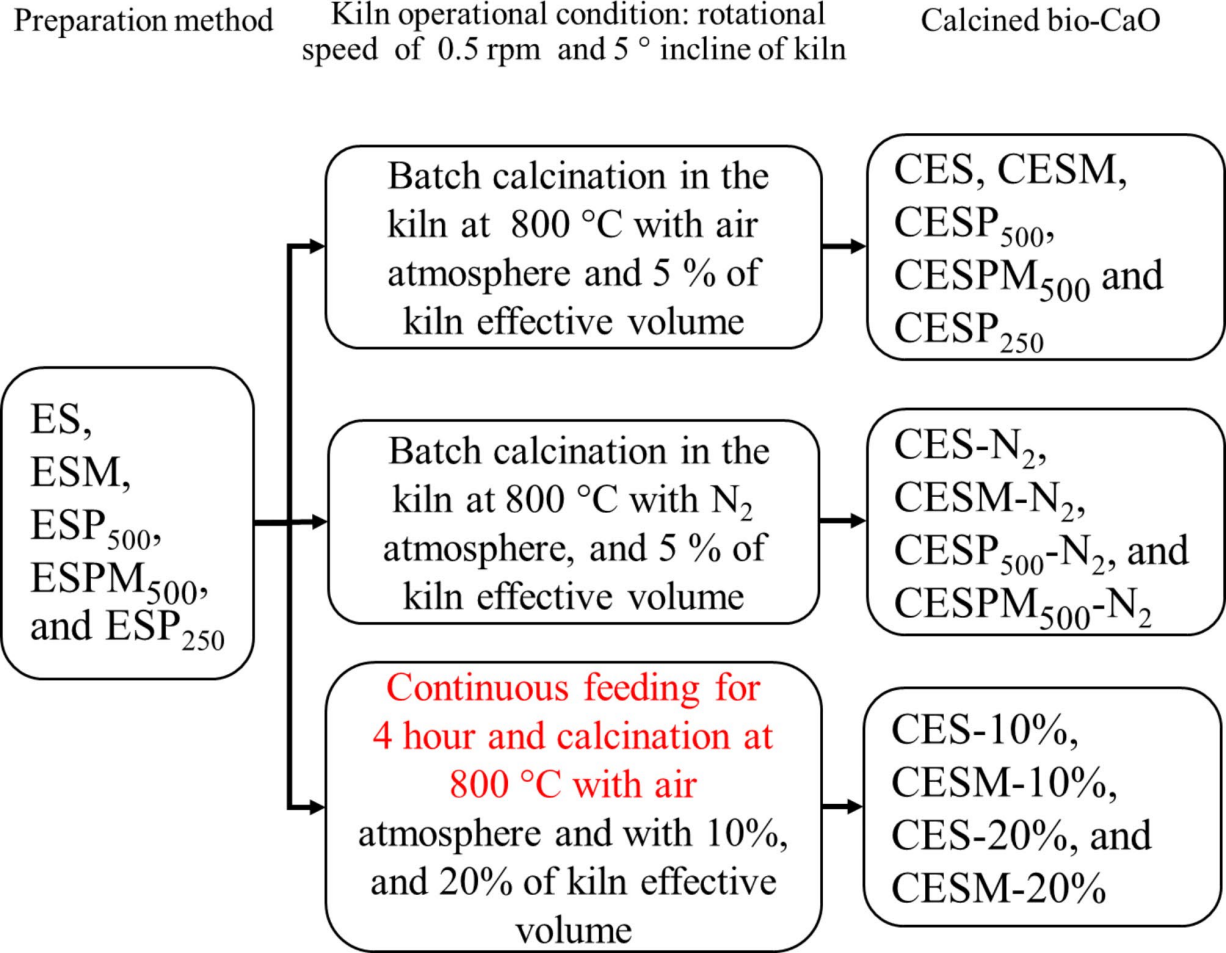
Flow diagram of calcination treatments and sample names.

The sample was continuously fed to the kiln for 4 h to determine the production stability and consistency of the product’s properties. Mixture of the calcined eggshells at the exiting of the kiln were collected from the 1st to 4th hour of operating time with the stainless hopper and kept in desiccator before further characterization. Summary of these treatments are shown in Fig. 4.

### Characterization of the samples

The following instruments were employed for the characterization of the eggshell waste and calcined eggshell waste samples. Color of the samples was determined by Konica Minolta Chroma Meter (CR-400). The microstructure of the samples was observed by a Field Emission Scanning Electron Microscope (FE-SEM Thermo Scientific ApreoS). Surface area and porosity analyzer (Tristar II plus) was used for determining surface area, pore volume, and mean pore size of the samples. The crystalline structure of the samples was examined by X-ray powder diffraction (XRD - PW 3040/60 X’PERT PRO Console) using Cu-Kα radiation at 40 kV. The XRD patterns of the samples were recorded with a scanning rate of 2 ° min^−1^ at 2θangles ranging from 5° to 80 °. The X-ray fluorescence (XRF Bruker S4 Explorer) was used for the analysis of the elemental composition of the samples. Heavy metals contained in the samples were analyzed using inductively coupled plasma mass spectroscopy (ICP-MS/OES Perkin Avio550). Analysis of loss on ignition, acid insoluble matter, and magnesium and alkali salts were determined using the guidelines of The Joint FAO/WHO Expert Committee on Food Additives (JECFA)^30,–31^.

## Results and discussion

### Morphology of the bio-CaO

**Fig. 5:**
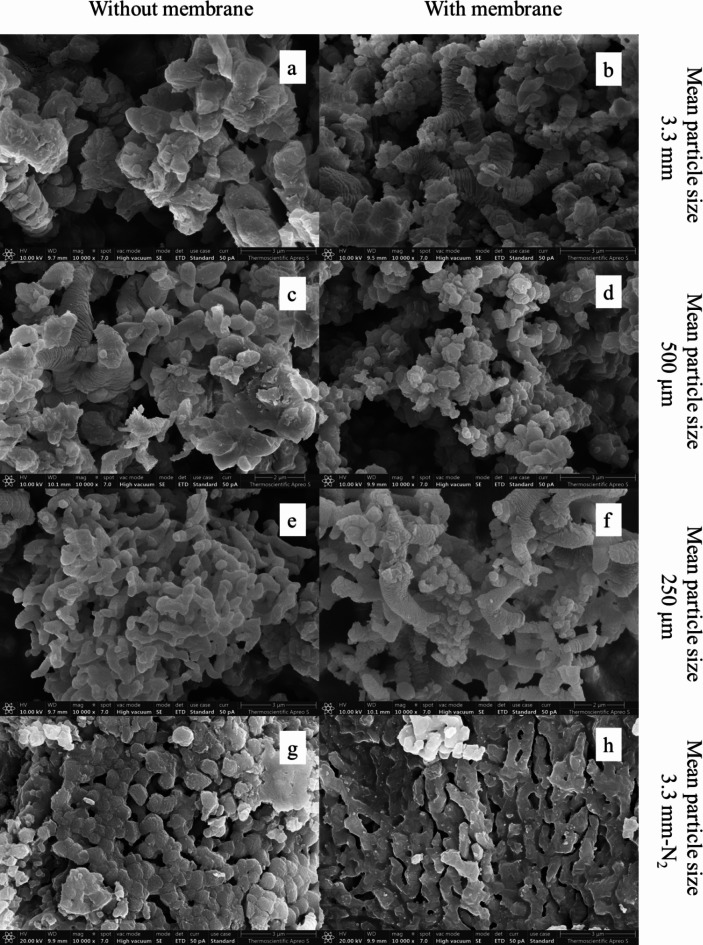
FS-SEM images of the bio-CaO obtained from different treatments (**a**) CES, (**b**) CESM, (**c**) CESP_500_, (**d**) CESPM_500_ (**e**) CESP_250_ (**f**) CESPM_250_. (**g**) CES-N_2_, (**h**) CESM-N_2_.

High resolution field-emission scanning electron microscopy (FE-SEM) images with ×10,000 magnification of the obtained bio-CaO prepared using different treatments and calcined at 800 °C with 0.5 rpm and with 5% kiln effective volume feeding rate are shown in Fig. 5. According to previous studies of the surface structure of the eggshells, decomposition of CaCO_3_ to CaO and CO_2_ in all samples at 800 °C changed the apparent morphologies of the eggshell surface structure from a smooth surface with some small pores to a porous structure. This is because of the decomposition of CaCO_3_ in the eggshell structure to CO_2_and CaO. There is obviously no eggshell membrane left in the calcined samples (CESM and CESP_500_), which were derived from eggshell waste containing eggshell membrane. This is a result of thermal decomposition of eggshell membrane in the temperature range of 400–600 °C, which is in accordance with previous reports^26^. However, all bio-CaO products obtained from different treatments show similar morphology of CaO particles containing both rod and unsymmetrical particle forms. Size of the bio-CaO particles observed by FS-SEM is in the range of 2–5 μm. In comparison, surface morphology of bio-CaO calcined in the N_2_ atmosphere (CES-N_2_ and CESM-N_2_) were less porous than the bio-CaO calcined in the air atmosphere. This is probably due to calcination of eggshell at 800 °C is not enough for completing CaCO_3_ decomposition to CaO and CO_2_.

### Resident time, yield, and color of the bio-CaO

As mentioned in calcination section, the calculated residence time of the samples in the heating zone of the rotary kiln is ~69 min. Besides this, the actual resident time of the eggshell samples was recorded when the samples initially entered and exited the heating zone. The result shows that the mean resident time of the eggshell sample with and without eggshell membrane was 71 ± 3.7 min and 72 ± 1.5 min, respectively. The obtained resident time is not much different from the calculated data. Meanwhile, the mean resident time of the eggshell-powder sample with and without eggshell membrane was 79 ± 1.4 min and 78 ± 2.2 min, respectively. It should be noticed that the powder samples with round shapes had slightly longer resident time than the ordinary samples with irregular shapes. Salengke and Sastry^32^ reported that the residence time of particles in the kiln decreased with a decrease in the particle size. Shimosaka et al.^33^ mobility of particles reduced with increasing irregularity shape of the particle. However, the phenomenon found in this study is possibly controlled by the heating condition instead of the particle size and shape of the samples. Valverde et al.^34^ found that short time preheating, or a high heating rate of the furnace leads to the formation of calcined powder agglomerate which sticks to the surface of the furnace and reduces their mobility.

The summary of yield and color characteristics of the samples and the bio-CaO products obtained via calcination in air and N_2_ atmospheres are shown in Tables 1 and 2, respectively. Production yield is one of the crucial factors for determining the possibility of a scalable production process. This is because low production yield may lead to high production costs and low beneficial return. In this study, the yield of the obtained bio-CaO product was determined by weighting the sample prior to and after the experiment. Additionally, all treatments were repeated three times to ensure the uncertainty of the experiment.

As shown in Table 1, the yield of the CaO product obtained from eggshells (CES) and eggshells containing eggshell membrane (CESM) was 54.9% and 51.8%, respectively. In comparison with the previous reports^19,35^, thermal decomposition of the eggshells yielded total mass loss in the range of 44–51 wt% depending on calcination temperature in the range of 795–1000 °C and calcium carbonate content in the eggshells. Additionally, Kristl et al.^36^ reported that the calculated theoretical mass loss of calcium carbonate decomposition is 44%. Thus, the production yield of calcium oxide was estimated to be about 49–56 wt%, which is similar to this study.

**Table 1:** Summary of yield and color characteristics of the eggshell sample and the bio-CaO obtained from calcination in the air atmosphere.

Sample	Yield (%)	L*	a*	b*	SCI	Apparent color
Raw eggshell	-	87.3 ± 0.09	1.44 ± 0.03	9.6 ± 0.22	76.3	Brown
Laboratory grade CaO	-	96.8 ± 0.00	-0.10 ± 0.00	1.8 ± 0.00	95.1	White
Industrial grade CaO	-	93.0 ± 0.40	-0.03 ± 0.00	2.5 ± 0.10	90.5	Light gray
Batch calcination at 800 °C for 1 h with air atmosphere, 0.5 RPM and 5% filling volume of kiln						
CES	54.9% ± 0.01	97.6 ± 0.1	0.00 ± 0.01	1.3 ± 0.02	96.3	White
CESM	51.8% ± 0.01	96.3 ± 0.4	0.03 ± 0.01	1.3 ± 0.04	95.0	White
CESP_500_	44.2% ± 0.00	97.5 ± 0.1	0.02 ± 0.04	1.4 ± 0.06	96.1	White
CESPM_500_	41.8% ± 0.02	96.2 ± 0.2	0.04 ± 0.03	1.2 ± 0.01	95.0	White
CESP_250_	37.2% ± 0.06	97.6 ± 0.1	0.03 ± 0.01	1.3 ± 0.03	96.3	White

Beside this, both calcined products CESP_500_ and CESPM_500_ derived from the 500 μm powder were obtained with a yield of 44.2% and 41.8%, respectively. Additionally, the CESP_250_ product was obtained in a much lower yield than the samples mentioned earlier. These results show that the calcined samples made from eggshell waste containing eggshell membrane yielded about 2.1–3.8% less product than samples made after eggshell membrane removal. This corresponds to the thermal decomposition of the membrane and turns into ash during the formation of both the CESM and CESPM_500_ products. Similar reports on the complete thermal decomposition of the eggshell membrane in the range of 500–600 ° C were also found in Putkham et al.^26^ and Cree et al.^37^. It should be noted that the large particle size of the raw materials used to produce calcined samples CES resulted in yields that were 10.7% and 7.7% higher than for the powder samples CESP_500_ and CESP_250_, respectively. This is probably due to the formation of a calcined powder sample agglomerate, which sticks to the surface of the furnace. This phenomena is similar to the observations made by Valverde et al.^34^, who reported that agglomeration of CaO particles is probably due to natural lime entering directly into the high temperature kiln without preheating or with fast heating rate of the kiln. Overall, it can be clearly seen that both eggshell membranes contained in the sample and size of raw materials play an important role in the production yield of bio-CaO. Furthermore, the CESP_250_ sample was not used for further study of the effect of N_2_ atmosphere since it gave the lowest yield of the calcined products.

The yields of bio-CaO derived from calcination in N_2_ atmosphere show a similar trend to the previous calcination treatment in the air atmosphere. The CES-N_2_ product, derived from raw materials with large particle size and with eggshell membrane removal, gave the highest calcined production yield (51.81%), as shown in Table 2. The calcined samples CES-N_2_ and CESP_500_-N_2_ also had the production yield higher than products CESM-N_2_ (2.65%) and CESPM_500_-N_2_ (2.80%), which were derived from the eggshell membrane containing raw materials. As shown in Table 2, the raw material feeding rate was increased in the next experiment from 5 to 10% and 20% of the kiln effective volume in order to determine the effect of feeding rate on the calcination product. The experiments CES-10% and CES-20% provided the CaO product in 54.65% and 54.40% yield, respectively. This demonstrates that the yield of bio-CaO did not decrease with the increase of the feeding rate from 5 to 20%. However, the calcined products CESM-10% and CESM-20%, which were derived from the eggshell membrane containing raw materials, still gave a lower value of production yield in the range of 2.3–2.9% than products CES-10% and CES-20%. It is noticed that these low production yields of CESM-10% and CESM-20%, due to the thermal decomposition of the membrane in the sample, agree well with the previous calcination treatment in the air atmosphere.

**Table 2:** Summary of yield and color characteristics of the bio-CaO obtained from calcination in N_2_ with 5% filling volume and in air atmosphere with the variation of raw material filling volume.

Sample	Yield (%)	L*	a*	b*	SCI	Apparent color
Batch calcination at 800 °C for 1 h with N_2_ atmosphere, 0.5 RPM and 5% filling volume						
CES-N_2_	51.85% ± 0.01	80.3 ± 0.06	0.24 ± 0.03	2.4 ± 0.10	77.7	Light gray
CESM-N_2_	49.20% ± 0.00	69.6 ± 0.71	0.27 ± 0.02	2.4 ± 0.03	66.9	Dark gray
CESP_500_-N_2_	44.30% ± 0.00	78.2 ± 0.76	0.25 ± 0.02	2.4 ± 0.06	75.6	Light gray
CESPM_500_-N_2_	41.50% ± 0.00	69.5 ± 0.15	0.29 ± 0.03	2.5 ± 0.02	66.7	Dark gray
Continuous calcination at 800 °C for 4 h with air atmosphere, 0.5 RPM and various filling volume						
CES-10%	54.65% ± 0.03	97.3 ± 0.2	-0.19 ± 0.1	1.1 ± 0.1	96.4	White
CES-20%	54.40% ± 0.01	96.5 ± 0.2	-0.07 ± 0.0	1.2 ± 0.0	95.4	White
CESM-10%	52.30% ± 0.00	96.5 ± 0.2	-0.13 ± 0.0	1.1 ± 0.1	95.5	White
CESM-20%	51.45% ± 0.00	96.4 ± 0.1	-0.13 ± 0.0	1.4 ± 0.0	95.1	White

In conclusion, the eggshell membrane and particle size of the eggshell play an important role in the production yield of bio-CaO. The yield of bio-CaO decreases with a decrease in the particle size of eggshell materials. Furthermore, calcination of the eggshell sample containing membrane also leads to a slightly low production yield of bio-CaO. However, calcination of the eggshell in either an air or nitrogen atmosphere does not show any significant effect on the yield of the CaO.

Color assessment of the bio-CaO is an important property of the filler as it may change the apparent color and could lead to an unpleasant color of the final product. A spectrophotometer (Konica Minolta CR-400) provides the lightness (L*), the red-green coordinate or redness (a*), and the blue-yellow coordinate or yellowness (b*)values of the samples as classified by the Commission International de L’Eclairage (CIE)^38^. The probe of the spectrophotometer was placed on the samples area and the L*, a*, and b* measurements were conducted in triplicate on the same sample. Subsequently, both mean values and standard deviation values were calculated. The mean values of L*, a *, and b* were used to calculate the color index of the eggshell (SCI) which is defined as: $\:SCI={L}^{*}-{a}^{*}-{b}^{*}$, where lower SCI values correspond to a darker color^39^.

Apparent color means and standard deviations of the L*, a*, b* parameters, and SCI values of the samples are reported in Table 1. The lightness (L*) values for the obtained bio-CaO calcite fabricated at 800 °C in air atmosphere have the average values in the range of 96.3–97.6. In these samples, the average values of redness (a*) were close to zero, whereas the yellowness (b*) values ranged between 1.2 and 1.4. Additionally, the SCI value of these samples was not significantly different, which indicates that neither eggshell membrane separation nor size of the eggshell raw material had any effect on the lightness color of the obtained bio-CaO. In comparison, very high SCI value of the bio-CaO shows that color of the obtained CaO is whiter than the raw eggshell and industrial grade CaO. This is probably due to the pigments present in the eggshells being completely decomposed at 800 °C^39^. In addition, the white color of the bio-CaO product is comparable to laboratory grade CaO.

The eggshells treated with different methods were also calcined at 800 °C under N_2_ atmosphere to determine the effect of the inert gas on the properties of bio-CaO. The results show that the average values of a* were in the range of 0.24–0.29, whereas the b* values ranged between 1.2 and 1.4. In addition, the L* values of these samples ranged between 69.5 and 80.3. This indicates that the obtained bio-CaO samples calcined in an N_2_ atmosphere have darker color than bio-CaO samples calcined in air atmosphere. Likewise, the SCI values obtained for bio-CaO calcined in N_2_ atmosphere also show similar tends to the L* values. This is probably because the pigments in the eggshells are more completely thermally decomposed in the oxidizing air atmosphere. Furthermore, the color of the CaO derived from the eggshell samples containing eggshell membrane (CESM-N_2_ andCESPM_500_-N_2_) tends to be darker than for the CaO derived from the eggshells alone (CES-N_2_ andCESP_500_-N_2_). It is apparent that soot may form during the decomposition of eggshell membrane and lead to a darker CaO product.

In this study, the calcination was performed with the percentage of raw material filling in the kiln set at 10% and 20% of kiln effective volume in an air atmosphere in order to determine the scalability of the production of the bio-CaO in the rotary kiln. The L*, a*, b* and SCI values of the corresponding bio-CaO products were also in the range of white color, which is similar to the color values of bio-CaO obtained with calcinations conducted using raw material filling rate of 5%of the effective kiln volume. However, the SCI value of the CaO derived from the eggshell alone (CES-10% and CES-20%) indicated a slightly whiter color than for the eggshell samples containing eggshell membrane (CESM-10% and CESM-20%). As mentioned earlier, this effect of membrane removal on the color of the obtained calcium oxide is consistent to the bio-CaO derived from calcination of eggshells in the N_2_ atmosphere. In summary, the increase of the material filling volume from 5 to 20% of the kiln effective volume slightly reduced the SCI color index of the bio-CaO product. This slightly lower SCI color index is possibly due to an increase in the material filling volume, which reduces efficient heat transfer to the eggshell, resulting in an incomplete decomposition of the pigment in the eggshell. Therefore, the design of the rotary kiln with lifters^40^ probably increases heat transfer and increases SCI color index.

### Surface area and pore volume

**Fig. 6:**
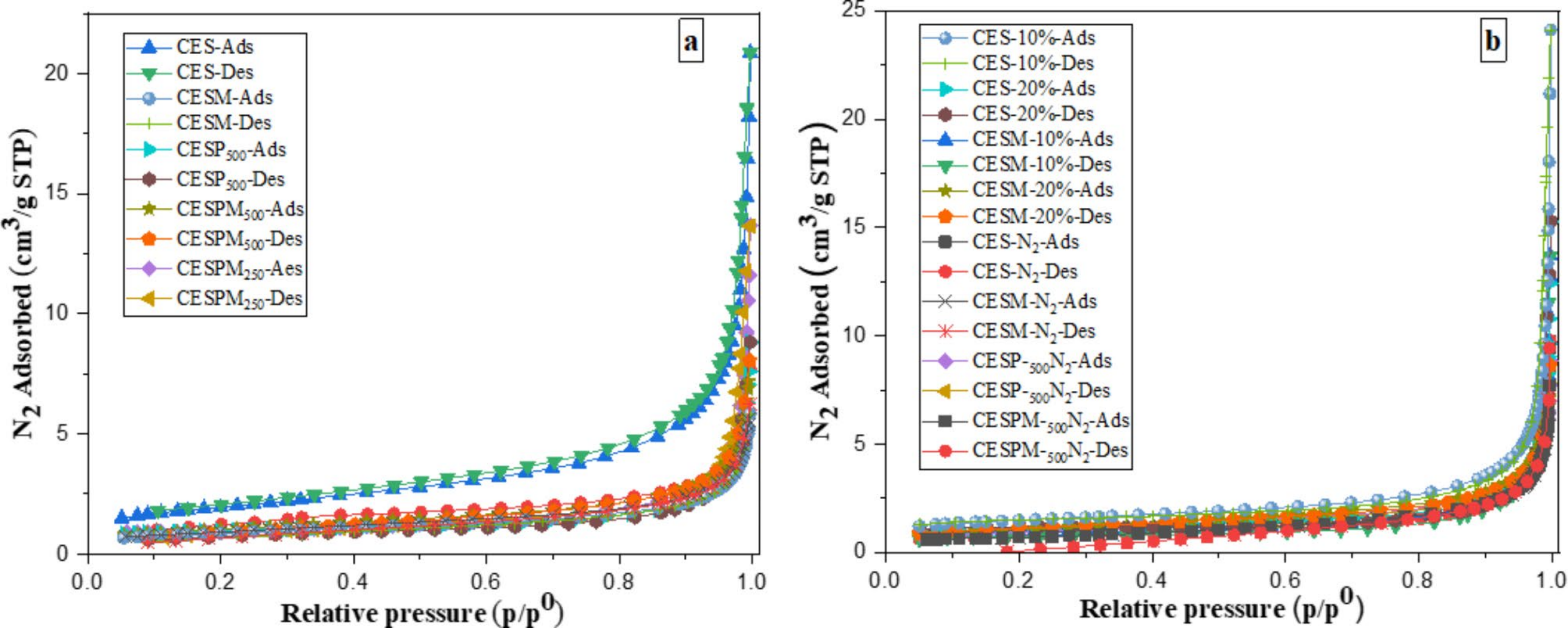
Adsorption and desorption isotherms of N_2_ on the bio-CaO products obtained from (**a**) different particle size preparation method with calcination in air atmosphere and 5% filling volume of the kiln and (**b**) calcination of eggshells in N_2_ atmosphere with 5% filling volume and calcination of eggshells in air atmosphere with filling volume of 10% and 20% of the kiln.

The surface area, pore volume, and pore size of a bio-CaO material has a direct impact on its catalytic activity. Adsorption and desorption isotherms of N_2_ on the bio-CaO products obtained from various treatments measured at −196 °C are shown in Fig. 6(a, b). All isotherms exhibited Type III characteristics according to the International Union of Pure and Applied Chemistry (IUPAC) classification scheme and no hysteresis loop is observed in these isotherms. These type III isotherms indicate weak adsorbate-adsorbent interactions and it should be noted that type III isotherms most commonly occur in both non-porous and macroporous adsorbents^41,42^.

Surface area, pore volume, and average pore size of bio-CaO products is shown in Table 3. Specific surface areas calculated by the Brunauer-Emmett-Teller (BET)^43^ method and pore volumes of the bio-CaO products derived from calcination at 800 °C for 1 h in an air atmosphere with 5% filling volume of kiln are relatively low and lie in the range of 3.07–6.88 m^3^/g and 0.008–0.028 cm^3^/g, respectively. The pore diameter values of the bio-CaO samples are in the range of mesopore or in the range of 20 Å − 500 Å. However, only the isotherm of the CES material prepared from calcination of ES with 3.3 mm mean particle size and without the eggshell membrane showed slightly higher N_2_ adsorption. In Addition, the CES product also has a slightly higher BET surface area (6.88 m^3^/g) and slightly higher pore volume (0.028cm^3^/g) than products made with other treatments. Beside this, both surface areas and pore volumes of the bio-CaO products obtained from the treatment of eggshell waste by calcination in an N_2_ atmosphere had similar values to the bio-CaO products obtained from the treatment of eggshell waste by calcination in an air atmosphere. These results show that the effect of either eggshell membrane removal or particle size of raw materials have a small effect on the surface areas and pore volumes of the bio-CaO products. Moreover, conducting the calcination with increasing raw material filling from 5 to 20% volume of the kiln also resulted in only a small effect on the surface areas and pore volumes of the obtained bio-CaO products. In comparison, the BET surface area of the CES product was found to be similar to a previous study^44,45^, which reported that BET surface area of raw eggshells is in the range of 2.33-6.34m^3^/g. Sharma et al.^46^ reported that CaO derived from eggshell has low total pore volume of 0.00722 cm^3^/g with 190 Å mesopore diameter. However, Pornchai et al.^25,45^ and Han et al.^36^ found that their calcined eggshell derived CaO had a BET surface area of 14.9 m^3^/g and 19.9 m^3^/g, respectively. The low BET surface areas and low pore volumes of these bio-CaO products might be due to the effect of long calcination times at a higher temperature, which leads to shrinkage of the pores of calcium oxide^11,34,47^.

**Table 3:** Surface area, pore volume and average pore size of bio-CaO obtained from various treatments.

Sample	BET surface area (m^2^ /g)	Pore volume (cm^3^ /g)	Micropore volume (cm^3^ /g)	Pore size (Å)
Batch calcination at 800 °C with air atmosphere, 0.5 RPM and 5% filling volume of kiln				
CES	6.88	0.028	0.0003	164
CESM	3.08	0.018	0.0010	153
CESP_500_	3.19	0.011	0.0007	150
CESPM_500_	3.67	0.011	0.0001	124
CESP_250_	3.07	0.008	0.0001	108
Batch calcination at 800 °C with N_2_ atmosphere, 0.5 RPM and 5% filling volume				
CES-N_2_	3.30	0.008	0.0001	106
CESM-N_2_	2.38	0.010	0.0003	175
CESP_500_-N_2_	3.44	0.010	0.0003	124
CESPM_500_-N_2_	3.31	0.012	0.0007	175
Continuous calcination at 800 °C for 4 h with air atmosphere, 0.5 RPM and various filling volume				
CES-10%	4.96	0.027	0.0007	222
CES-20%	3.61	0.015	0.0007	175
CESM-10%	3.14	0.016	0.0007	209
CESM-20%	3.77	0.010	0.0003	112

### XRD analysis

**Fig. 7:**
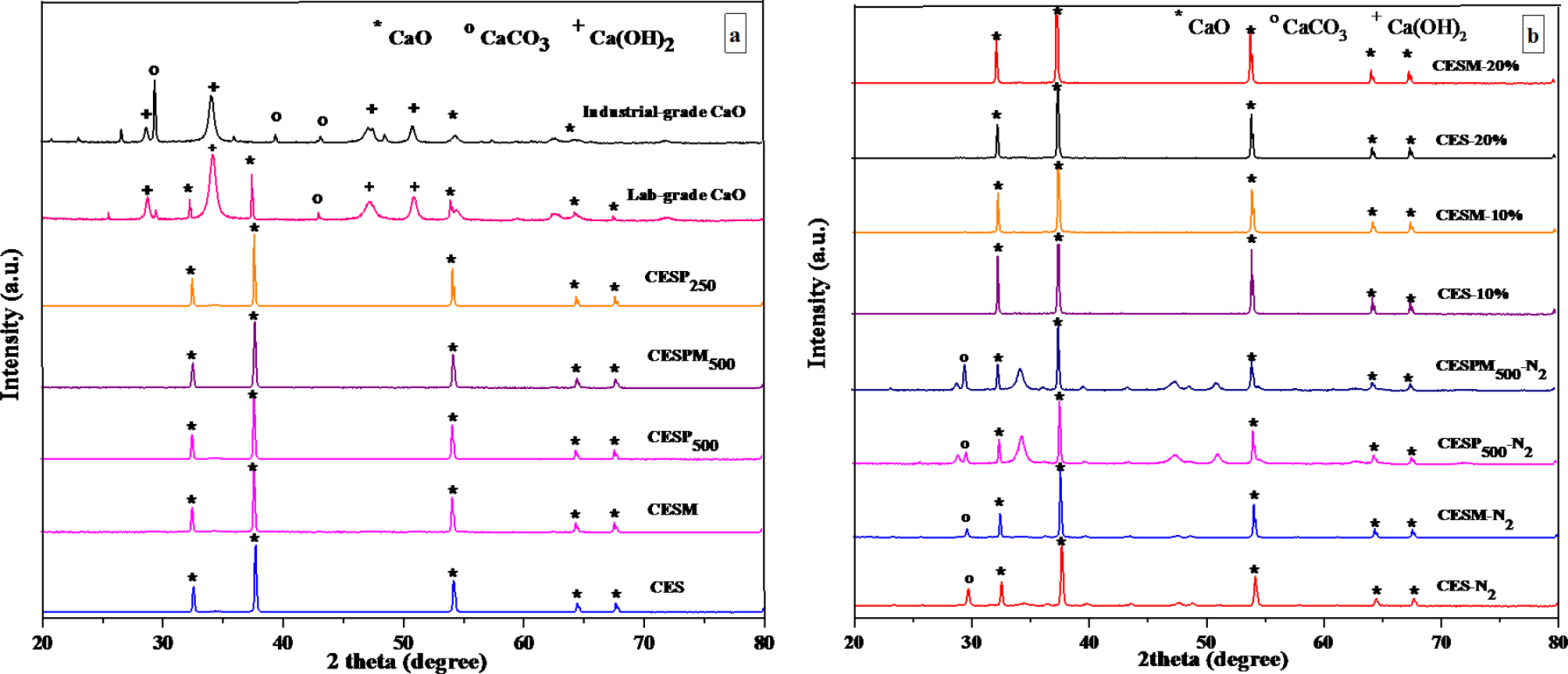
The XRD patterns of bio-CaO products obtained from (**a**) different particle size preparation method calcined in the air atmosphere and with a 5% feeding rate and (**b**) calcination of eggshells either in N_2_ atmosphere with 5% filling volume or calcinations of eggshells in air atmosphere with filling volume of 10% and 20% of the kiln.

The comparisons of XRD patterns of industrial CaO, laboratory grade CaO, and the bio-CaO derived from various treatments conducted in this work are shown Fig. 7(a, b). As shown in Fig. 7a, the XRD results reveal that the bio-CaO samples obtained from both CES and CESM starting materials calcined in the air atmosphere with 5% filling volume were found to be composted of CaO (*2θ* = 34.0°, 50.7°, 62.5°, and 71.7°) as their XRD diffractograms matched well with a standard diffractogram of a calcium oxide of the Joint Committee on Powder Diffraction Standards (JCPDS).Furthermore, the XRD patterns of powder samples CESP_500_, CESPM_500_, and CESP_250_ also reveal similarities of crystalline peaks when compared to the pattern of standard CaO provided by the JCPDS data. It is worth noting that neither differences in particle size or eggshell membrane removal had any effect on the crystal structure of the bio-CaO product. For the industrial grade CaO, the main peak was observed at *2θ* = 29.0° and other peaks were present at *2θ* = 36.0°, 39.0°, 44.0°, 47.0°, and 48.0°. These peak values are indicative of the presence of CaCO_3_. In comparison, the peaks CaCO_3_present in the industrial grade CaO were not present in the bio-CaO products. These differences in the XRD profile of the bio-CaO products are caused by complete thermal decomposition of CaCO_3_ in the eggshells to CaO and CO_2_.

Figure 7b shows the XRD patterns of CaO derived from either calcination carried out in N_2_atmosphere with 5% feeding rate or calcination carried out in air atmosphere with the variation of raw material filling rate. The XRD results show that both crushed samples (CES-N_2_ and CESM-N_2_) and powder samples (CESP_500_-N_2_ and CESPM_500_-N_2_) calcined in N_2_ atmosphere with 5% filling volume mainly consisted of CaO. However, CaCO_3_ is present in these products as evidenced by medium intensity peaks of CaO_3_ (*2θ* = 36.0°, 39.0°, 44.0°, 47.0°, and 48.0°). This is attributed to the fact that the calcined eggshells were not totally converted to CaO. It is clear that calcination of the eggshell samples in N_2_ atmosphere at 800 °C is not sufficient to completely decompose CaCO_3_. These results are similar to the observation of Razali, et al.^48^ who reported that the optimum temperature for calcination of chicken eggshell waste in an inert atmosphere is in the range of 850–900 °C. Figure 7b also shows the XRD patterns of the samples calcined in the air atmosphere with feeding rate of 10% and 20%. The XRD patterns of the samples without eggshell membrane (CES-10% and CES-20%) show intense peaks of CaO at *2θ* = 32.2°, 37.3°, 53.8°, 64.2°, and 67.5 and these peaks also align with the spectrum of standard CaO. Similar XRD patterns were observed for the samples with eggshell membrane (CESM-10% and CESM 20%). It is obvious that the presence of the eggshell membrane in the raw materials has no effect on the crystal structure of the obtained CaO products. Moreover, increasing the filling volume of the kiln from 5 to 20% also did not alter the crystal structure of the CaO product. These observations agree well with previous findings about the CaO product with high SCI color index.

### Chemical composition of the samples

Chemical composition of the obtained CaO products compared to various samples and Thailand industrial standard institute (TISI) is shown in Table 4. Based on the XRF analysis, bio-CaO content in the samples derived from the CES, CESP_500_, and CESP_250_ products calcined in the air atmosphere with 5% filling volume was 98.1%, 98.0%, and 97.9%, respectively. Similarly, bio-CaO content in the CESM and CESPM_500_ products was 97.1% and 97.0%, respectively. In addition to CaO, there are five major trace components present in the bio-CaO samples which were in the range of 1.03–1.20% for MgO, 0.29–0.35% for P_2_O_5_, 0.16–0.35% for SO_3_, 0.43–0.44% for SrO, and 0.027–0.032% for SiO_2_. Under these circumstances, the purity of these five calcined samples conformed to the Thailand industrial standard institute (TISI 319). It is clear that particle size of the raw samples does not show an effect on the purity of the CaO products while the purity of the CaO obtained from eggshells containing eggshell membrane was reduced by about 1%. This implies that the removal of the eggshell membrane from the raw eggshell waste is not necessary to produce an industrial grade CaO. This new finding can probably lead to an alternative process to reduce the production costs of bio-CaO from eggshell waste. In comparison, the percentage of bio-CaO in the products obtained from this study was like in previous reports^49,50^, which found that the purity of CaO obtained from calcination of eggshell waste is in the range of 97–98%.

**Table 4:** Chemical composition of the obtained CaO products derived from different treatments and Thailand industrial standard institute 319 (TISI).

Composition	CaO	MgO	SO_3_	*P*_2_O_5_	Na_2_O	K_2_O	SrO	SiO_2_	ZrO_2_	Al_2_O_3_	Fe_2_O_3_	
CES	98.1	1.03	0.291	0.356	0.102	0.014	0.044	0.032	0.002	–		–
CESM	96.9	1.2.0	0.352	0.716	0.329	0.104	0.043	0.027	0.001	–	–	
CESP_500_	98.0	1.04	0.161	0.353	0.248	0.048	0.043	0.031	0.001	–	–	
CESPM_500_	97.0	1.27	0.348	0.742	0.373	0.114	0.044	-	0.001	–	–	
CESP_250_	97.9	1.13	0.189	0.404	0.137	0.025	0.046	0.081	0.001	–	0.018	
CES-N_2_	96.7	1.26	0.862	0.579	0.151	0.040	0.043	0.309	0.002	–	0.018	
CESM-N_2_	96.0	1.29	1.460	0.757	0.176	0.072	0.043	0.137	0.002	–	0.015	
CESP_500_-N_2_	96.2	1.32	1.890	0.794	0.200	0.070	0.046	0.174	0.001	–	0.020	
CESPM_500_-N_2_	94.9	1.38	1.510	0.894	0.250	0.075	0.048	0.14	0.001	–	0.023	
CES-10%	97.9	1.22	0.208	0.535	0.090	0.018	0.047	0.045	0.001	–	0.016	
CES-20%	97.8	1.27	0.172	0.392	0.144	0.024	0.045	0.112	0.001	–	0.018	
CESM-10%	97.3	1.13	0.189	0.404	0.137	0.025	0.046	0.081	0.001	–	0.018	
CESM-20%	97.3	1.21	0.528	0.528	0.150	0.028	0.047	0.084	0.001	–	0.018	
Raw eggshell^51^	96.1	0.53	0.599	0.283	–	0.188	0.305	0.504	–	0.353	0.177	
Calcined eggshell^51^	97.0	054	0.123	0.316	–	0.103	0.263	1.266	–	0.255	0.013	
Industrial grade CaO	94.3	1.51	0.273	0.363	–	0.019	2.9	–	0.17	0.033	0.009	
Laboratory grade CaO	98.2	0.66	0.150	0.030	–	0.570	0.050	0.140	0.040	0.080	0.009	
TISI 319 Grade I	≥ 90	≤ 1.8	≤ 0.5	≤ 0.4	≤ 1.0	–	–	–	–	–	–	

Table 4 also shows chemical composition of the obtained CaO products derived from either calcination in N_2_ with 5% kiln volume filling or air atmosphere with variation of kiln filling volume. The XRF analysis shows that content of the bio-CaO in the samples derived from both CES-N_2_ and CESP_500_-N_2_ calcined in the N_2_ atmosphere with 5% kiln filling volume was 96.7% and 96.2%, respectively. In the same way, the content of bio-CaO in the CESM-N_2_ and CESPM_500_-N_2_products is 96.0% and 94.9%, respectively. Besides that, five major trace components found in the bio-CaO samples are MgO, SO_3,_ P_2_O_5,_ SrO, and SiO_2_. This is similar to the major trace components found in the bio-CaO obtained from calcined eggshell waste in air atmosphere. The XRF analysis also shows that the particle size of the starting samples does not have an effect on the purity of the CaO products. However, the purity of the CaO obtained from eggshell waste containing eggshell membrane was reduced by about 0.7–1.3%. This slightly lower CaO content is consistent with the low SCI color index found in the previous section for these materials. The slightly lower CaO content might be caused by an incomplete decomposition of the CaCO_3_ in the eggshells.

The effect of further increasing the kiln filling volume and membrane removal on the purity of the obtained CaO is also shown in Table 4. The XRF analysis shows that the content of CaO present in the samples is 97.9% for CES-10%, 97.8% for CES-20%, 97.5% CESM-10%, and 97.3% for CESM-20%. Similarly, increasing the kiln filling volume from 5 to 20% shows an insignificant effect on the purity of the obtained bio-CaO samples. However, purity of the CaO obtained from eggshell waste containing eggshell membrane was reduced by approximately 0.5–0.6% but the purity of the bio-CaO product from this kiln filling volume treatment still conforms to the TIS standard. This small amount of ash membrane found in this study is consistent with the previous reports. Mensah et al.^51^ pointed out that the eggshell membrane contains about 80–85% of protein, carbohydrate, and fat while 10–15% of the membrane is trace inorganic elements. Nakano et al.^52^ reported that the ash content in the eggshell membrane is about 0.31 ± 0.05%. Thus, the membrane removal process might not be required for the production of bio-CaO with the rotary kiln. Furthermore, these results also demonstrate that large-scale continuous production of CaO is possible.

### Specifications for food additives

The International Numbering System for Food Additives (INS) assigns CaO as the code INS 529 and classifies it as a food additive with the potential functions of altering and controlling the acidity or alkalinity of food. The Joint FAO/WHO Expert Committee on Food Additives (JECFA)^30^ announced the specification of food grade CaO as shown in Table 5. Both European Union (EU) and Thailand Food and drug administration (FDA) also adopt this JECFA standard to control the specification of food grade CaO^53^. The comparison of the bio-calcium products CES and CEMS with the JECFA standard using the mean values with standard deviations is shown in Table 5. It is clear that the specifications of the CES and CEMS samples meet the JECFA standard. The chemical content of impurities in the CEMS product, which was obtained from eggshells containing eggshell membrane, was slightly higher than for the CES samples. These results correspond well with the XRF data. This indicates that the impurities might have originated from the eggshell membrane. In addition, lead was not detected neither in the CES nor in the CEMS sample.

**Table 5:** Comparison of bio-CaO specification obtained from eggshell and specification of CaO for food additive announced by JECFA.

Parameters	Sample		JECFA (CaO)
	CES	CESM	
Assay	98.1	97.1	≥ 95.0%
Description	Pass	Pass	Odourless, hard, white, or greyish white masses of granules, or white to greyish powder
Loss on ignition	0.9 ± 0.1	1.6 ± 0.6	≤ 10% (At 800 °C)
Acid insoluble matter	0.2 ± 0.1	0.4 ± 0.5	≤ 1%
Magnesium and alkali salts (mg)	0.4 ± 0.03	0.6 ± 0.18	≤ 3.6%
Barium (mg/kg)	6.3 ± 0.2	5.7 ± 0.1	≤ 300 mg/kg
Arsenic (mg/kg)	0.06 ± 0.02	0.08 ± 0.03	≤ 3 mg/kg
Lead (mg/kg)	Not Detected	Not Detected	≤ 2 mg/kg

## Conclusion

Factors effecting the operational production of bio-CaO derived from hatchery eggshell waste via thermal decomposition in a rotary kiln are reported for the first time in this study. All calcination treatments were carried out at 800 °C with a rotational speed of 0.5 RPM and a 5° inclination of the kiln. This study found that the preparation methods of raw eggshell samples play an important role in determining the purity of the obtained CaO. Production yield of CaO is increased by about 17.7% upon increasing the particle size of raw eggshell waste from 250 μm to 3.3 mm. The purity of the obtained CaO decreased by about 0.7–1.3% when the calcination is carried out with eggshell waste containing eggshell membrane. The color assessment and SCI index indicated that calcination of samples in the air atmosphere provides a preferable white color of the bio-CaO product while calcination in the N_2_ atmosphere provides the CaO product with gray to dark gray color. The XRD and XRF analyses showed that all the obtained bio-CaO products were crystalline and the purity of the obtained CaO products was in the range of 96–98%. The specific BET surface area of this mesoporous bio-CaO was in the range of 3.07–6.88 m^3^/g. This study also found that further increasing of raw material filling in the kiln from 5 to 20% has only slightly altered both production yield and purity of the obtained CaO. This implies that optimum continuous production of CaO with this rotary kiln is possible on a large scale. Furthermore, the purity of the bio-CaO produced from these experiments conforms to both an industrial standard and a food additive standard.

## Electronic supplementary material

Below is the link to the electronic supplementary material.

Supplementary Material 1

## Data Availability

The datasets used and/or analyzed during the current study available from the corresponding author on reasonable request.
