# Supplementary material for: Scalable production of bio-calcium oxide via thermal decomposition of solid - hatchery waste in a laboratory-scale rotary kiln
Source: Sci Rep. 2025 Jan 5;15:865. doi: 10.1038/s41598-024-84889-w (PMC11701074; doi:10.1038/s41598-024-84889-w)
Supplement: Supplementary file 1 — Supplementary Material 1 [file 41598_2024_84889_MOESM1_ESM.pdf]

Scalable Production of Bio-Calcium Oxide via Thermal Decomposition of Solid - Hatchery Waste in a Laboratory-Scale Rotary Kiln

Suwanan Chuakham[†], Ajchara I. Putkham[‡], Yuwadee Chaiyachet[†] Arnusorn Saengprajak[⁂], Kriangsak Banlue[‡‡], Nipon Tanpaiboonkul[†], and Apipong Putkham*,[†]

[†]Department of Environmental Technology, Mahasarakham University, Mahasarakham 44150, Thailand

[‡]Department of Chemistry, Naresuan University, Phitsanulok 65000, Thailand

[⁂]Department of Physics, Faculty of Science, Mahasarakham University, Mahasarakham 44150, Thailand

[‡‡]Department of Food Technology and Nutrition, Faculty of Technology, Mahasarakham University, Mahasarakham 44150, Thailand

Supplementary material

This indirect heating rotary kiln is made of quartz reactor tube which is 1,200 mm long and has a 440 mm effective heating zone. Before conducting the experiment, the PID-controlled heaters were programmed to heat the quartz reactor to 920 °C and was equilibrated for 1 hour. Then, the temperature inside the quartz tube chamber was measured directly using the type K thermocouples. The temperature distribution along the quartz reactor of the kiln is shown in Fig.S1. The shape of the temperature curves was divided to three zones inside the reactor. The average temperature in the heating zone is about 810 ± 4.2 °C.

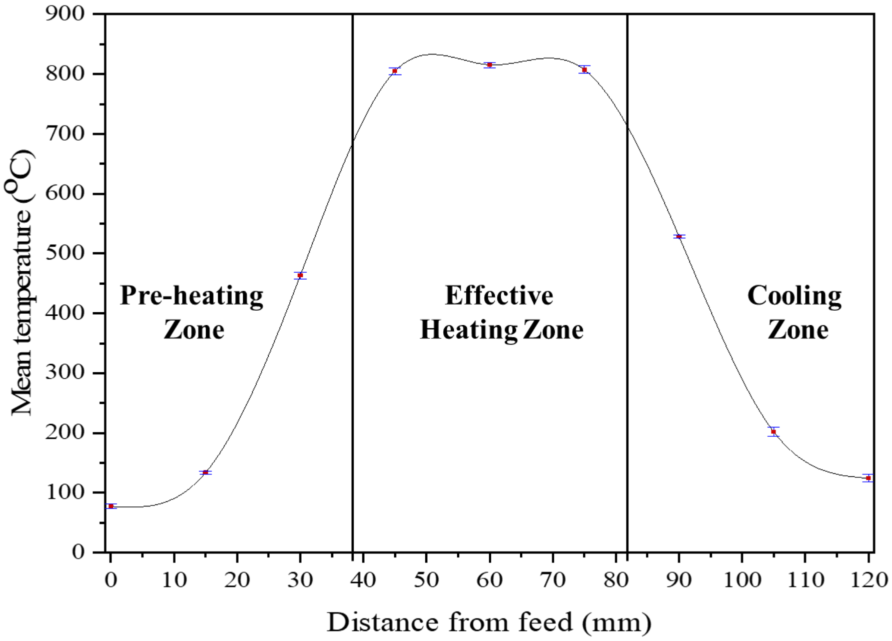

Fig.S1. The average temperature profile inside the rotary kiln.
